# The Audience-Tuning Effect of Negative Stereotypes in Communication

**DOI:** 10.3389/fpsyg.2021.663814

**Published:** 2021-07-20

**Authors:** Junhui Ye, Lei Zhao, Zijuan Huang, Fanxing Meng

**Affiliations:** ^1^School of Marxism, Zhejiang Police College, Hangzhou, China; ^2^Research Institute of Applied Psychology, Zhejiang Police College, Hangzhou, China; ^3^School of Management, Zhejiang University of Technology, Hangzhou, China; ^4^Department of Psychology and Behavior Science, Zhejiang University, Hangzhou, China; ^5^Department of Criminal Investigation, Zhejiang Police College, Hangzhou, China

**Keywords:** audience-tuning, communication, memory-tuning, shared reality, stereotypes

## Abstract

Shared reality theory states that people allow others to influence their own judgments and behaviors when a shared reality is achieved (Hardin and Higgins, 1996; Echterhoff et al., 2009a). Based on this theory, this research has explored how audience attitude affects the communicator’s memory of negative stereotype-related information in interpersonal communication. Two experiments have been conducted, using the negative stereotypes of Chinese “rich second-generation” as the research materials. The results show that the audience-tuning effect of negative stereotypes does in fact occur in interpersonal communication. The participants have tuned their descriptions of both stereotype-related and neutral information to suit their audience’s attitude toward the target. The audience-tuning affects the participants’ recall valence of stereotype-related information while not affecting the recall valence of neutral information. The relational motivation moderates the effect of audience-tuning on the communicator’s memory of stereotype-related information. Only participants who communicated with a desired audience displayed an audience-congruent memory bias of stereotype-related information. The results of this research reveal the bidirectional nature of stereotype-sharedness in interpersonal communication. In actual interpersonal communication, the audience could express a positive attitude toward the target who suffers from negative stereotypes, and the communicator would then convey and recall the stereotype-related information in a more positive manner based on the audience-tunning effect, which could ultimately help to decrease negative stereotypes in communication.

## Introduction

Stereotypes are commonplace in our lives. Examples of stereotypes include: women are bad at science, men are bad in humanities, Chinese Americans are cold and unsociable, etc. Stereotypes are a cognitive structure formed by an individual’s fixed ideas or expectations about members of a specific group (Fiske, 2004). The content of stereotypes comprises our understandings, beliefs, and expectations of certain social groups (Hamilton et al., 1990). Stereotypes can help us quickly classify and process complex social information. However, they can also cause prejudice and discrimination of certain groups, which may lead to serious social problems.

Many stereotypes that people have do not come from their own direct experience but are obtained from interpersonal communication (Karasawa et al., 2007). For example, patients transmit their own stereotypes about doctors to others (Wang et al., 2018). Through platforms such as the Internet, mass media has been able to deliver stereotypes to a broad audience (Wang and Sun, 2005). In the social learning process of stereotypes, communication plays an important role. Interpersonal communication contributes to stereotype processing, transformation, and maintenance (Ruscher and Hammer, 2006). Lots of stereotype-related information is disseminated in interpersonal communication. If an individual’s negative stereotype about a specific group is accepted by most members of society through interpersonal communication, then a collective negative stereotype of this target group will emerge, which will evolve into prejudice.

Researchers have conducted many studies on interpersonal communication of stereotypes both in oral and written communication (Kashima, 2000; Lyons and Kashima, 2003; Clark and Kashima, 2007; Kurylo and Robles, 2015; Robles and Kurylo, 2017). For instance, Kurylo and Robles (2015) conducted a qualitative research on undergraduates’ responses to interpersonally oral communicated stereotypes. They found that the communicators had at least 13 ways of responding to stereotypes. Most of these response types were not in opposition to the stereotypes but rather in tolerance of the stereotypes in interactions. Lyons and Kashima (2003) explored the stereotype sharedness in written communication by using the method of serial reproduction to simulate a chain of interpersonal communication. In their study, four participants formed a serial reproduction chain. The first participant in the chain read a given story and reproduced the story in writing for the second participant to read. The story was related to stereotypes of a target group and contained an equal number of stereotype-consistent sentences and stereotype-inconsistent sentences. The second participant read the first participant’s reproduction and reproduced it to the third participant in the same way and so on until the last participant finished the reproduction. The analysis of the reproductions indicated that when the communicator believed that most of the group members endorsed the stereotype, the communicator would share more stereotype-consistent information. Another study also showed that the stereotype consistency bias not only existed in a communication chain composed of multiple people but also in communication between two people (Clark and Kashima, 2007). These studies revealed that people were inclined to communicate more stereotype-consistent information than stereotype-inconsistent information. This selective sharing of stereotype-related information showed that communicators would adjust their communication content of stereotypes according to the social situation. Communication facilitates the dissemination and maintenance of stereotypes. As the communicator shares more stereotype-consistent information, more stereotype-consistent information is received by the audience. Thus, the communicator contributes to the maintenance of stereotypes within an audience through communication (Lyons and Kashima, 2003). However, communication is a process in which the communicator and the audience influence each other. Previous studies have mainly focused on how the communicator influences the audience’s information processing of stereotypes. Conversely, might the communicator’s information processing of stereotypes be influenced by the audience in interpersonal communication? Specifically, how does the audience’s attitude affect the communicator’s sharing of content and their subsequent memory of stereotype-related information?

### Audience-Tuning Effect and the Shared-Reality Theory

Researchers have found the audience-tuning effect in dyadic communication. Communicators would adjust their communication content according to the characteristics of the audience (e.g., personality, intention, attitude, etc.) (Echterhoff et al., 2008, 2009b). For example, in Higgins and Rholes’s (1978) study, participants were asked to first read pieces of information about a target person with an ambiguous character (could be either positive or negative) before describing the target (without mentioning the name) to an audience who liked/disliked the target. Meanwhile, the participants were informed that the audience’s task was to identify the target person from a group of people based on the participant’s description. It was found that participants adjusted their descriptions to be congruent with the audience’s attitude. The participants who communicated to an audience who liked the target described the target more positively. Meanwhile, the participants who communicated to an audience who disliked the target described the target more negatively. Audience-tuning can promote the effectiveness of communication by ensuring that the information is more understandable (Fussell and Krauss, 1989) and persuasive (Higgins, 1981). Furthermore, previous studies have also found that audience-tuning would change the communicator’s subsequent memory of the target person, resulting in an audience-congruent bias. It is the memory-tuning effect, or the “saying-is-believing” effect (SIB effect) (Ding and Zheng, 2011). The audience-tuning effect on memory can persist from a few minutes to a few days.

The shared-reality theory explains the underlying mechanism of the SIB effect. The central idea of the theory suggests that shared reality is the product of the process in which individuals experience a commonality of inner states with others (Echterhoff et al., 2009a). When people experience uncertainty or ambiguity, it is very necessary for them to achieve a shared reality with others. Festinger (1950) pointed out that people seek the social reality provided by others when facing ambiguous situations. Once shared reality with others is reached, an individual’s judgment and behavior start to be influenced by the inner state of others, as they are trusted as a source of valid information. Shared reality is driven by fundamental human needs and motives, specifically the need to understand the world and connect with others (Echterhoff et al., 2009b; Kopietz et al., 2010).

The SIB effect occurs when the communicators create a shared reality with their audience about the target person. The inner state sharing causes the communicators to experience a certain aspect of the target in common with the audience. Thus, the communicators may tune their descriptions and memories of the target according to their audience’s attitude (Ding and Zheng, 2011). The audience-tuning and memory-tuning effects have been found in studies on religious beliefs (Magee and Hardin, 2010), group member betrayal (Mannetti et al., 2010), interpersonal affiliation (Huntsinger and Sinclair, 2010), and many other fields. For example, in a study on eyewitness memory, the researchers found that the eyewitness’s memory was influenced by the co-witness’s impression of the suspect. In the experimental task, the participants watched an ambiguous forensic video. Then, they had to describe the event depicted in the video to a co-witness (their audience) and issue a penalty for the suspect in the video. The co-witness attitude toward the suspect elicited an audience-congruent bias on the witness’s memory and judgment (Kopietz et al., 2009).

The establishment of shared reality plays an important role in effective communication (Kashima et al., 2010). For instance, some researchers have identified the phenomenon of interactive alignment in verbal communication. According to the Interactive Alignment Model (IAM) proposed by Pickering and Garrod (2004), communicators automatically align on one another’s linguistic representations at multiple levels (e.g., phonological, lexical, and syntactic). Such interactive alignment is assumed to underlie successful mutual understanding or the achievement of a “common ground” between interlocutors during communication. Thus, this interactive alignment could also be seen as a shared reality between interlocutors. These studies all indicate that people tend to affiliate and establish a connection with others. This desire for connectedness prompts people to tailor the information they wish to deliver to be congruent with the audience’s attitude in communication, thus achieving a shared reality with the audience. Therefore, we expect audience-tuning of negative stereotypes to also occur in communication. We propose the following hypothesis:

H1: Participants tune their descriptions of negative stereotypes and neutral information to their audience’s attitude toward the target.

Stereotypes have certain processing advantages in memory processing compared to neutral information. Due to the limited capacity of memory load, individuals tend to prioritize processing the more important, valence information (e.g., negative information). Stereotype-based expectancies facilitate the processing of stereotype-related information (Hamilton et al., 1990). Participants recall stereotype-consistent information better than stereotype-irrelevant information (Rothbart et al., 1979; Stangor and Ruble, 1989). Thus, we suggest that the type of information may affect the memory-tuning effect. The memory-tuning effect of neutral information may not be as significant as the effect of negative stereotypes. The hypothesis is proposed as:

H2: Negative stereotypes have a greater memory-tuning effect than neutral information.

### Relational Motivation

Echterhoff et al. (2009a) pointed out that the state of shared reality can only be achieved with the existence of underlying motivations. It is one of the four necessary conditions that underlie shared reality. Previous studies have demonstrated that the influence of the audience-tuning on communicators’ memory is driven by motivation (Echterhoff et al., 2005, 2009b; Pierucci et al., 2013, 2014). Specifically, shared reality has a certain degree of selectivity, and people are more willing to share reality with the people they like. That is, relational motivation affects the occurrence of shared reality in communication.

Relational motivation refers to the motivation that drives people to connect with others in the pursuit of happiness and a sense of belonging, thereby strengthening their self-identity or self-esteem (Echterhoff et al., 2009a). For example, people have higher relational needs toward their ingroup members compared to outgroup members (Echterhoff et al., 2008). Some researchers have found that only participants who communicated with the ingroup audiences had an audience-congruent biased memory. However, there was no audience-congruent memory bias for the participants who communicated with the outgroup audiences (Echterhoff et al., 2005). Pierucci et al. (2013) manipulated their participant’s desire to communicate with the audience through the audience selection procedure. They found that only participants who communicated with their desirable audience exhibited the audience-congruent memory bias. These results reveal the important role of relational motivation for shared reality. We expect relational motivation also to affect the audience-tuning effect of negative stereotypes on the memory. Only when communicating with the desired audience do the participants tune their memory of negative stereotypes, to be congruent with their audiences. We are proposing the following hypothesis:

H3: Relational motivation moderates the audience-tuning effect on the memory of negative stereotypes.

We are also formulating the following sub-hypotheses:

H3a: After communicating with a desired audience, the participants will exhibit an audience-congruent memory bias.

H3b: After communicating with an undesired audience, the participants will exhibit no audience-congruent memory bias.

## Pilot Study

The purpose of the pilot study is to conduct research material for the formal study. In recent years, China’s unique development background has led to a huge gap between the rich and the poor. Individuals belonging to the “rich second-generation,” who were born with silver spoons in their mouths, are viewed by the public in a very negative light. This group is often reported negatively by the Chinese media for their arrogance and spendthrift ways. Surveys have also shown that negative attitudes toward the “rich second-generation” group continue to exist in current Chinese society (Xie, 2012; Yin et al., 2013). Thus, we decided to use the negative stereotypes of Chinese “rich second-generation” as the research material in our studies. First, we verified the presence of existing negative stereotypes concerning this group. We recruited 109 undergraduates and required them to describe “rich second-generation” college students and ordinary college students using 10 adjectives. The results showed that the proportion of negative adjectives for “rich second-generation” was significantly higher compared to ordinary college students [χ^2^ (2, *N* = 1878) = 171.39, *p* < 0.001] as well as positive [χ^2^ (1, *N* = 681) = 60.51, *p* < 0.001] and neutral adjectives [χ^2^ (1, *N* = 664) = 72.89, *p* < 0.001]. This confirms that there are indeed negative stereotypes of the “rich second-generation” group.

Based on the SIB effect paradigm (Echterhoff et al., 2005), we used the adjectives obtained from the survey above as the behavior descriptions of the target person. These behavior descriptions were ambiguous and could be understood as either positive or negative characteristics. To compare the difference between the audience-tuning of stereotype-related information and the neutral information, we also constructed neutral behavior descriptions of the target person. The research material contained six behavior descriptions, of which three were related to stereotypes of the “rich second-generation” (e.g., Li Ming spends a lot of time doing things that excite him. Without professional training, he has gone trekking, surfing, and kayaking. This statement can be perceived as either rash or adventurous). The other three descriptions were neutral information (e.g., Li Ming has always been very meticulous. Even when taking notes, he pays attention to his handwriting and the organization of the notes. However, he often fails to complete tasks on time because of his attention to detail. It can be perceived as either pedantic or meticulous).

To verify the validity of the research materials, we recruited another 108 participants and divided them into two groups to evaluate the valence of these six behavior descriptions of the target person (seven-point Likert scale, −3 to 3, *very negative* to *very positive*). The target person of one group was labeled as the “rich second-generation” college student, and the other group was labeled as an ordinary college student. We found that when the target person was an ordinary college student, participants’ evaluations of these six behavior descriptions were all neutral (close to 0), which verified the ambiguity of the research materials. The evaluations of the stereotype-related information (SR information) of the target person with the “rich second-generation” label were significantly more negative than those of the ordinary college students [*t* (106) = 2.28, *p* = 0.02, Cohen’s *d* = 0.44], while there was no difference in the neutral information [*t* (106) = 0.17, *p* = 0.86, Cohen’s *d* = 0.03], indicating that SR information was closely related to the “rich second-generation” stereotypes.

## Experiment 1

Experiment 1 adopted the experimental paradigm of the SIB effect (Higgins and Rholes, 1978) to explore the audience-tuning effect of negative stereotypes on interpersonal communication. According to the shared-reality theory, we inferred that audience-tuning and memory-tuning of negative stereotypes occurred in communication.

### Methods

#### Participants

Forty college students from Zhejiang University were recruited to participate in the experiment, receiving payment (RMB 15 for each participant) as a reward. The data from one participant was deleted due to incomplete answers. Thus, the data from 23 women and 16 men (mean age = 19.72 years, *SD* = 1.12) were analyzed.

#### Design

A single-factor between-subjects experimental design was adopted. The independent variable was the audience’s attitude toward the target person (like/dislike). All participants were randomly assigned to one of the two experimental conditions. The dependent variables were the valence of the description messages and recall texts.

#### Procedure

The experiment task required participants to describe the target person to an audience. Participants were informed that the audience would identify the target person based on their description. All experimental tasks were completed by computer. First, participants were asked to read an essay about the target person (a “rich second-generation” named Li Ming). The essay consisted of six paragraphs, of which three paragraphs were neutral ambiguous behavior descriptions (neutral information), and the other three were ambiguous behavior descriptions related to stereotypes (SR information). After that, participants were informed about their audience’s attitude toward the target person (like/dislike):

“As a result of knowing Li Ming personally, the audience has developed their own impression of Li Ming: Our previous observations indicate that the audience actually seems to like [doesn’t seem to like] Li Ming and believes that Li Ming has [does not have] many good qualities.”

Then participants were asked to enter their descriptions of the target person into the text box and send it to their audience. Participants were required to abstain from mentioning the target person’s name in their descriptions. Afterward, participants were told that it would take a certain amount of time for the audience to give feedback, during which time the participants were asked to complete an unrelated elimination game task. After 10 min of the unrelated task, participants were informed that the audience had successfully identified the target person through their descriptions, and the participants were then asked to recall their original descriptions of the target person.

#### Ethics Statement

All participants provided written informed consent before participating in the experiments. The participants were reminded of their right to discontinue participation at any time. The Research Ethics Board of Zhejiang University approved all procedures.

#### Measures

Each description and recall text was broken down into units according to the original passages of the target information. Two experts in the field of stereotypes went through all the units and independently divided them into two categories (SR information/neutral information). The two experts discussed their differences before reaching an agreement. Then, two psychology graduate students blind to the experimental conditions scored the valence of each unit on an 11-point Likert scale, ranging from −5 (*very negative*) to +5 (*very positive*). Descriptions and recall message units were presented in random order to the coders. Correlations between the coders’ ratings were sufficiently high (*r*s = 0.78 and 0.77 for the valence of SR information and neutral information, respectively, *p*s < 0.001). The average scores of the two coders served as the dependent measures.

### Results and Discussion

#### Audience-Tuning

**FIGURE 1:**
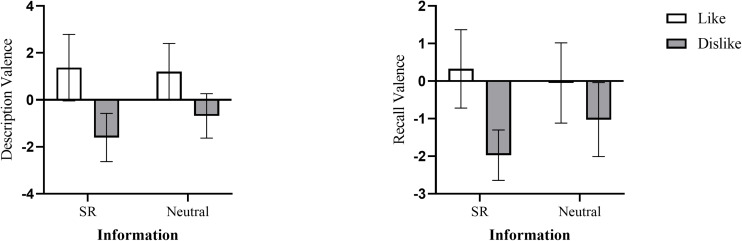
Description valence (**left panel**) and recall valence (**right panel**) as a function of the audience’s attitude and the information (bars represent 95% confidence intervals).

In order to compare the differences in audience-tuning between the two types of information, we conducted an ANOVA on the interaction between information and audience attitudes. The results showed the main effect of the audience’s attitude was significant, *F* (1,37) = 11.76, *p* = 0.001, η*^2^_*p*_* = 0.24. Neither an effect of information [*F* (1,37) = 1.13, *p* = 0.30, η*^2^_*p*_* = 0.03] nor an interaction [*F* (1,37) = 2.44, *p* = 0.13, η*^2^_*p*_* = 0.06] were found. Further analysis found a simple effect of the audience’s attitude for SR information, *F* (1,37) = 12.52, *p* = 0.001. For SR information, participants described the target more positively for the audience who liked the target (*M* = 1.38, *SD* = 3.03) than for the audience who disliked the target (*M* = −1.61, *SD* = 2.13). Similarly, a simple effect of the audience’s attitude for neutral information was found, *F* (1,37) = 12.52, *p* = 0.001. For neutral information, participants also described the target more positively for the audience who liked the target (*M* = 1.20, *SD* = 2.58) than for the audience who dislike the target (*M* = −0.68, *SD* = 1.97) (see Table 1 and Figure 1). Thus, we consider H1 verified. Participants tuned both descriptions of SR information and neutral information messages according to their audience’s attitude. Audience-tuning occurred for both SR information and neutral information in interpersonal communication.

**TABLE 1:** Description valence and recall valence as a function of information and audience attitude.

**Information**	**Audience attitude**	**Description valence**		**Recall valence**	
		**M**	**SD**	**M**	**SD**
SR information	Like (*n* = 20)	1.38	3.03	0.33	2.23
	Dislike (*n* = 19)	−1.61	2.13	−1.97	1.39
Neutral information	Like (*n* = 20)	1.20	2.58	−0.05	2.28
	Dislike (*n* = 19)	−0.68	1.97	−1.03	2.04

#### Audience-Tuning Effect on Memory

We also conducted an ANOVA on the interaction between information and audience attitudes to compare the differences in memory-tuning between the two types of information. The results showed the main effect of the audience’s attitude was significant, *F* (1,37) = 10.47, *p* = 0.003, η*^2^_*p*_* = 0.22. Neither an effect of information [*F* (1,37) = 0.50, *p* = 0.49, η*^2^_*p*_* = 0.01] nor an interaction [*F* (1,37) = 2.66, *p* = 0.11, η*^2^_*p*_* = 0.06] were found. Further analysis found a simple effect of the audience’s attitude for SR information, *F* (1,37) = 14.78, *p* < 0.001. For SR information, participants recalled more positive characters about the target when their audience liked the target (*M* = 0.33, *SD* = 2.23) than when their audience disliked the target (*M* = −1.97, *SD* = 1.39). But there was no significant difference in the recall valence of neutral information, *F* (1,37) = 1.97, *p* = 0.17 (see Table 1 and Figure 1). Our results indicated the audience-tuning only affected participants’ recall of SR information. Thus, H2 regarding negative stereotypes have a greater memory-tuning effect than neutral information was verified.

In summary, the results of Experiment 1 indicated that the ambiguous behavior descriptions influence the participants to adjust their descriptions of the target person according to the audience’s attitude. The audience-tuning effect would occur for both SR information and neutral information. However, in the recall stage, the audience-tuning had different effects on the recall of SR and neutral information. The memory bias only occurred for SR information.

## Experiment 2

According to the shared-reality theory, relational motivation is an important factor affecting the establishment of shared reality. In Experiment 2, we manipulated the participants’ desire to communicate with their audience to explore the influence of relational motivation on the audience-tuning effect of negative stereotypes in communication.

### Materials and Methods

#### Participants

Ninety-one college students from Zhejiang University were recruited to participate in the experiment, with payment (RMB 15 for each participant) being received as a reward. We deleted the data of 11 participants whose relational motivation we failed to manipulate, including the participants who were unwilling to communicate with the audience (the willingness to communicate was less than 0) in the high relational motivation condition and the participants with a high willingness to communicate with the audience (the willingness to communicate was greater than 0) in the low relational motivation condition. The data from 51 women and 29 men (mean age = 20.39 years, *SD* = 1.42) were analyzed.

#### Design

The participants were randomly assigned to one of the conditions of a 2 (audience attitude: like/dislike) × 2 (relational motivation: high/low) between-subjects experimental design.

#### Procedure

The experiment procedure and tasks of Experiment 2 were similar to those of Experiment 1. The only difference was that after the participants had read the descriptive essay of the target person, we manipulated their level of relational motivation. Based on the research of Pierucci et al. (2013), we manipulated the participants’ willingness to communicate with their audience. Participants were presented with four photos of the audiences^1^, including the two most attractive photos (one each for man and woman) and the two most unattractive photos (one each for man and woman) (Li, 2008). Participants were asked to choose one of the photographed persons (with the same gender) they wanted to communicate with the most (high relational motivation condition) or the least (low relational motivation condition) to be their audience. Then participants were asked to rate their willingness to communicate with the chosen audience on an 11-point Likert scale, ranging from −5 (*very unwilling*) to +5 (*very willing*).

#### Measures

To obtain the valence scores of both description messages and recall texts, we used the same coding procedure as in Experiment 1. Coder intercorrelations were sufficiently high (*r*s = 0.73 and 0.69 for the valence of SR and neutral information, respectively, *p*s < 0.001), so mean scores for description and recall valence could be calculated.

### Results and Discussion

#### Audience-Tuning

For SR information, the results of the analysis of variance (ANOVA) showed that the main effect of the audience’s attitude was significant, *F* (1,76) = 13.91, *p* < 0.001, η*^2^_*p*_* = 0.15. Neither the effect of relational motivation [*F* (1,76) = 1.04, *p* = 0.31, η*^2^_*p*_* = 0.01] nor interaction [*F* (1,76) = 0.01, *p* = 0.93, η*^2^_*p*_* < 0.001] were found.

**FIGURE 2:**
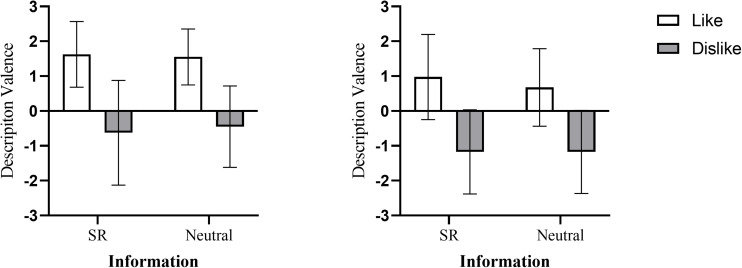
Description valence as a function of the audience’s attitude and the information under high relational motivation condition (**left panel**) and under low relational motivation condition (**right panel**) (bars represent 95% confidence intervals).

Similarly, for neutral information, the main effect of the audience’s attitude was significant, *F* (1,76) = 13.88, *p* < 0.001, η*^2^_*p*_* = 0.15. Neither the effect of relational motivation [*F* (1,76) = 2.40, *p* = 0.13, η*^2^_*p*_* = 0.03] nor interaction [*F* (1,76) = 0.02, *p* = 0.89, η*^2^_*p*_* < 0.001] were found (see Table 2 and Figure 2).

**TABLE 2:** Description valence as a function of relational motivation and audience attitude.

**Relational motivation**	**Audience attitude**	**Description valence**		**Description valence**	
		**of SR information**		**of neutral information**	
		**M**	**SD**	**M**	**SD**
High	Like (*n* = 20)	1.63	2.01	1.55	1.72
	Dislike (*n* = 20)	−0.63	3.21	−0.45	2.50
Low	Like (*n* = 20)	0.98	2.61	0.68	2.38
	Dislike (*n* = 20)	−1.18	2.58	−1.18	2.55

These results of Experiment 2 showed that the audience-tuning effect occurred for both SR information and neutral information, which was consistent with the results of Experiment 1. Regardless of the willingness to communicate with the audience, participants adjusted their descriptions of the target to the audience’s attitude toward the target person.

#### Audience-Tuning Effect on Memory

**FIGURE 3:**
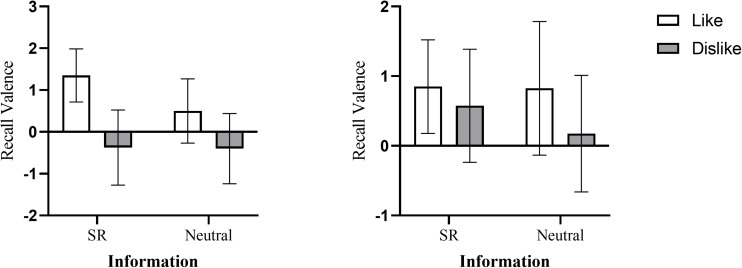
Recall valence as a function of the audience’s attitude and the information under high relational motivation condition (**left panel**) and under low relational motivation condition (**right panel**) (bars represent 95% confidence intervals).

For SR information, the results of the ANOVA showed a main effect exhibited by the audience’s attitude, *F* (1,76) = 7.56, *p* = 0.007, η*^2^_*p*_* = 0.09. The interaction between the audience’s attitude and relational motivation was also significant, *F* (1,76) = 3.97, *p* = 0.05, η*^2^_*p*_* = 0.05. Further analysis found a simple effect of the audience’s attitude in the high relational motivation condition, *F* (1,76) = 11.24, *p* = 0.001. After communicating with the desired audience, participants recalled the SR information in a more positively biased way when their audience liked the target (*M* = 1.35, *SD* = 1.36) than when their audience disliked the target (*M* = −0.38, *SD* = 1.92). The results supported H3a. However, there was no significant difference in the recall valence of SR information in the low relational motivation condition, *F* (1,76) = 0.29, *p* = 0.60 (see Table 3 and Figure 3). The results supported H3b.

**TABLE 3:** Recall valence as a function of relational motivation and audience attitude.

**Relational motivation**	**Audience attitude**	**Recall valence of SR information**		**Recall valence of neutral information**	
		**M**	**SD**	**M**	**SD**
High	Like (*n* = 20)	1.35	1.36	0.50	1.64
	Dislike (*n* = 20)	−0.38	1.92	−0.40	1.80
Low	Like (*n* = 20)	0.85	1.43	0.83	2.05
	Dislike (*n* = 20)	0.58	1.73	0.18	1.79

These results were consistent with the study of Pierucci et al. (2013). The memory-tuning effect only occurs in the high relational motivation condition. Thus, H3 was verified.

For neutral information, there were no significant effects of audience attitude [*F* (1,76) = 3.61, *p* = 0.06], relational motivation [*F* (1,76) = 1.22, *p* = 0.27] and interaction [*F* (1,76) = 0.09, *p* = 0.76]. Thus, the audience-tuning only affected the participants’ recall of SR information, which was consistent with the results of Experiment 1.

In summary, the results of Experiment 2 indicated that relational motivation does indeed affect the memory-tuning of SR information. The memory-tuning effect of negative stereotypes was found to only occur in the high relational motivation condition.

## General Discussion

Interpersonal communication plays an important role in the transmission and maintenance of stereotypes. Research in the past has shown that the audience receives more stereotype-consistent information from the communicator, which leads to the maintenance of the audience’s stereotypes. Most of this existing research focuses on the influence of the communicator on the formation of the audience’s stereotype. The present studies focus on the reverse process, namely, whether the audience’s characteristics (e.g., attitude, communication desire) affect the communicator’s memory of stereotypes information. To our knowledge, no studies have explored the effect of the audience on the stereotype processing of the communicator. Our studies can reveal the bidirectional influence of stereotypes in communication.

The results of our studies have shown that the communicator produced an audience-congruent description bias and an audience-congruent memory bias in social interaction. That is, a shared reality about negative stereotypes has been established between the audience and communicator. The audience’s attitude toward the target person affects the communicator’s memory of SR information. The results of our studies have also shown that relational motivation affects the memory-tuning of negative stereotype-related information. The memory-tuning effect of negative stereotypes only occurs in the high relational motivation condition. The results of the present studies enrich the communication and dissemination of stereotypes in the interpersonal context and reveal the bidirectional nature of stereotype sharedness.

In current studies, both Experiment 1 and Experiment 2 have shown that the audience-tuning effect and the memory-tuning effect of SR information occur in communication. This result has shown the stability of the stereotype sharedness, indicating that the communicator will share the audience’s psychological reality in the communication process and tailor the memory of SR information according to the audience’s attitude. This result is also consistent with the IAM, proving that, as communicators interact with each other, they dynamically adjust and adapt to each other, so that their cognition becomes gradually convergent during communication (Pickering and Garrod, 2004).

Higgins pointed out in his study that the communicator’s act of tuning information according to the audience’s attitude can be seen as labeling of the target person (Higgins and Rholes, 1978). The descriptions of the target persons in our studies consist of ambiguous information. However, our pilot study showed that when the target person was from the “rich second-generation” group, the individual’s evaluation of SR information was not neutral but more negative. Based on the current results, even the individual’s initial perception of the target has a certain tendency (either positive or negative), it does not prevent the individual from seeking social information to get a better understanding of the reality. Therefore, sharing of SR information can also occur as long as the descriptions of the target are ambiguous. The current results not only demonstrate the stability of stereotype sharedness, but also indicate that stereotype sharedness occurs to reduce uncertainty. This result is consistent with the findings of other studies on the impact of information ambiguity on a shared reality. A study on the judgment of suspected sexual harassment behavior also confirmed the role of information ambiguity in shared reality. When the participants were provided with testimonies about a supervisor’s ambiguous behavior toward a female employee, their memory was tuned according to the attitude of the co-witness (the audience). When other information references (such as information of eventual clear-cut harassment) were provided, participants did not tune their memory (Pierucci et al., 2014).

The results of Experiment 1 have shown that the ambiguous behavior descriptions influence participants to adjust their descriptions of the target person according to the audience’s attitude. The audience-tuning effect occurs for both SR information and neutral information. However, the audience-tuning has different effects on the recall of SR and neutral information in the recall stage. The memory bias only occurs on SR information, which might be due to the advantageous processing of SR information. Stereotype-based expectancies facilitate the processing of SR information (Hamilton et al., 1990). Some studies have demonstrated that participants recall stereotype-consistent information better than stereotype-irrelevant information (Rothbart et al., 1979; Stangor and Ruble, 1989). While some empirical evidence has indicated that participants recall stereotype-inconsistent information better than stereotype-consistent or irrelevant information (Srull and Wyer, 1989), participants will generally allocate the least attention to stereotype-irrelevant information (Plaks et al., 2001). Neutral information was processed less, as such information is irrelevant to the stereotype. Therefore, SR information is more easily retrieved in the recall phase compared to neutral information (Sherman et al., 1997). There is also no memory-tuning of neutral information in the recall stage. Therefore, SR information is an important reference for the communicator to form social perceptions in interpersonal communication.

Based on the shared-reality theory, relational motivation drives people to share reality with others. People gain a sense of connectedness through social sharing (Echterhoff et al., 2009a). Experiment 2 explored the influence of relational motivation on the audience-tuning and memory-tuning of negative stereotypes in communication. The memory-tuning effect of negative stereotypes only occurred in the high relational motivation condition. This result confirms the shared-reality theory in the context of stereotypes. When the audience’s attitude toward the target is positive, the communicator delivers the SR information in a more positively biased way. The communicator would even reconstruct the SR information in memory to make it more positive when communicating with the desired audience. However, the results are somewhat different from previous studies on stereotypes. Previous studies on the dissemination of stereotypes have also proven the phenomenon of stereotype-consistency bias (Ruscher and Duval, 1998). The situated functional model points out that people get a sense of social connection with others by delivering stereotype-consistent information (Clark and Kashima, 2007). However, most studies on stereotype-consistency bias did not take the audience’s characteristics (e.g., attitude, communication desire) into account. Our studies suggest that communicators actually pick up on their audience’s characteristics and take these characteristics into account during actual social interactions. The present studies indicate that, although the communicators may label the target negatively, the communicators would tailor the communication content and their subsequent memory of the stereotype to connect with their audience. Previous studies have mainly focused on how the communicator influences the audience’s information processing of stereotypes. The results of our studies suggest that the audience may influence the communicator’s information processing of stereotypes, as communication is a process in which the communicator and the audience influence each other. The present studies have, thus, revealed the bidirectional nature of stereotype sharedness. Our findings are also inconsistent with the IAM. IAM assumes that a primitive and resource-free priming mechanism achieves the interactive alignment. The communicators tend to automatically align their mental representations both for desired and non-desired audiences (Pickering and Garrod, 2004). However, according to our results, the tuning-effect is restricted to the desired audience.

In contrast to memory-tuning, Experiment 2 found that the relational motivation did not influence the audience-tuning effect. This is consistent with previous studies (Echterhoff et al., 2005; Echterhoff et al., 2009a; Kopietz et al., 2009). Communicators usually tune their message even to a disfavored audience. For instance, undergraduate participants tuned their descriptions not only to the ingroup audience (a student from the same university) but also to the outgroup audience (a trainee from a vocational school) (Echterhoff et al., 2005). However, Pierucci and his colleagues found that relational motivation does influence the audience-tuning effect. Audience-tuning only occurred for participants in the high relational motivation condition (Pierucci et al., 2013). This is consistent with self-presentation theory (Schlenker and Weigold, 1992). People may be more motivated to express thoughts and beliefs consistent with their audience to the extent that they wish to establish a positive relationship with this audience. Further research is needed to verify whether relational motivation does affect the tendency to exhibit audience-turning effects.

Negative stereotypes often lead to prejudice and even discrimination. In social interaction, the communication and dissemination of negative stereotypes often aggravate prejudice and discrimination (Fiske, 2000). How to suppress the influence of stereotypes on memory, especially that of negative stereotypes, plays an important role for people to better understand the world and improve their interpersonal relationships. However, researchers often encounter many difficulties in reducing and changing stereotypes, especially for a long term. The studies on the stereotype-consistency bias emphasize that individuals’ stereotypes are maintained through interpersonal communication. If people participate in the social world, their stereotypes are likely to be maintained as long as people around them omit the stereotype-inconsistent messages and retain the stereotype-consistent messages in their communication. By participating in the communication chain, communicators not only contribute to the maintenance of audience stereotypes but also to the collective maintenance of stereotypes with the stereotype-consistent information they spread interpersonally. In this sense, attempts to produce long-term stereotype change in particular individuals are always likely to fail as long as people continue participating in communities that share stereotypes (Lyons and Kashima, 2003). Our studies offer new perspectives on changing stereotypes through communication. The results of our research have revealed that stereotype sharedness in communication is a process in which the communicator and the audience influence each other. When faced with a desired audience, the communicator will share the audience’s positive attitude toward the target person and reconstruct the SR information in memory to make it more positive. Therefore, we can promote a shared reality between the communicator and the audience that holds a positive attitude, thereby changing the negative stereotypes that the communicator holds. While previous studies on audience-tuning have shown that the memory-tuning effect can last for several days (Higgins and Rholes, 1978). Future research needs to explore intervention strategies concerning negative stereotypes based on the audience-tuning effect and their influencing factors. Future studies can also focus on strengthening the memory-tuning effect of negative stereotypes so that it can persist over a longer period of time.

One possible limitation of current studies is the use of simulated computerized communication. Our studies have adopted the standard SIB paradigm in which the audience is a pure receiver of information. However, the audience is replaced by a computer in the experiment. Such a normative listener has no real information exchange with the communicator and no personality characteristics. Past research suggests that live audience feedback may lead communicators to feel more accountable to their audience (Lerner and Tetlock, 1999) and work harder to provide accurate information (Krauss and Fussell, 1996). This may affect the communicator’s processing of SR information. Future research could further improve the study of the audience-tuning effect on negative stereotypes by using a real audience. Another limitation is that we only use the Chinese “rich second-generation” as the stereotype target. The vast majority of studies related to stereotypes are based on natural cue social classification, such as sex, race, and age (Shutts et al., 2013; Weisman et al., 2015). In addition to natural cues, people also make social classification according to social cues (such as occupational role, political party, social status, etc.). For example, people tend to be classified into high and low social classes by their wealth (Kraus et al., 2012). The negative stereotypes of Chinese “rich second-generation” used in current studies are also social categories based on social cues. Whether our findings could extend to the natural cues remains unknown. However, since the shared reality between the communicator and the audience is only influenced by the ambiguity of the situation and their motivations (Echterhoff et al., 2009a), it is very likely that social classification (natural or social cues) do not affect the results of our studies, we could also found a similar audicen-tuning and memory-duning effect. Of course, future researches are needed to further investigate the audience-tuning effect of different negative stereotypes in communication and explore the robustness of this effect.

## Conclusion

The current studies have provided new insight into the stereotype sharedness within interpersonal communication. The results have shown that the dissemination of negative stereotypes undergoes the audience-tuning effect in communication. The audience’s attitude affects the communicator’s memory of SR information, but this memory-tuning effect occurs only when communicators communicate with a desired audience. The results of the current studies reveal the bidirectional nature of stereotype sharedness in communication and provide implications for stereotype intervention.

## Data Availability Statement

The original contributions presented in the study are included in the article/supplementary material, further inquiries can be directed to the corresponding author/s.

## Ethics Statement

The studies involving human participants were reviewed and approved by The Research Ethics Board of Zhejiang University. The patients/participants provided their written informed consent to participate in this study.

## Author Contributions

JY, LZ, and FM contributed to conception and design experiment and wrote the manuscript. ZH performed the experiment. JY, ZH, and FM analyzed the data. JY and LZ contributed equally to this work and share first authorship. All authors edited and/or approved the manuscript.

## Conflict of Interest

The authors declare that the research was conducted in the absence of any commercial or financial relationships that could be construed as a potential conflict of interest.

## Footnotes

1 All photos were selected from the experimental materials in Li (2008). Thirty-two subjects were rated according to the attractiveness of appearance, thereby distinguishing the high attraction group from the low attraction group. Two photos (one each for man and woman) were selected from the high attraction group and the low attraction group respectively. The four photos were used as the experimental materials for Experiment 2.

## Appendix

### Appendix A: Target Essay Used in Experiments 1 and 2 (Evaluatively Ambiguous Passages)

SR Information:

Li Ming spends a lot of time doing things that excite him. Without professional training, he has gone trekking, surfing, and kayaking (rash/adventurous).

Li Ming likes to talk to people, especially about his own interests. He can keep talking even if the other person shows no interest (nagging/sociable).

Li Ming has a direct communication style. One of his classmates discussed the homework with Li Ming and asked for some advice. He bluntly told the classmate that the homework was poorly done at the very beginning (offensive/outspoken).

Neutral Information:

Li Ming has always been very meticulous. Even when taking notes, he pays attention to his handwriting and the organization of the notes. However, he often fails to complete tasks on time because of his attention to detail (pedantic/meticulous).

Li Ming has recently become interested in European culture. He read books about Europe, attends Western music appreciation workshops, and eats in European style restaurants. When being with friends, he always talks a length about European culture and art (artificial/cultivated).

Many people appreciate Li Ming’s humor. He always likes to make some sudden jokes to make everyone happy. He would quickly seize the opportunity to make some jokes about others at parties (caustic/humorous).

Note. The word pairs in brackets at each paragraph end represent the two opposite trait labels that can be drawn from that paragraph.
